# Why the Mittag-Leffler Function Can Be Considered the Queen Function of the Fractional Calculus?

**DOI:** 10.3390/e22121359

**Published:** 2020-11-30

**Authors:** Francesco Mainardi

**Affiliations:** Dipartimento di Fisica e Astronomia, Università di Bologna, Via Irnerio 46, I-40126 Bologna, Italy; francesco.mainardi@bo.infn.it

**Keywords:** fractional calculus, Mittag-Lefflller functions, Wright functions, fractional relaxation, diffusion-wave equation, Laplace and Fourier transform, fractional Poisson process complex systems

## Abstract

In this survey we stress the importance of the higher transcendental Mittag-Leffler function in the framework of the Fractional Calculus. We first start with the analytical properties of the classical Mittag-Leffler function as derived from being the solution of the simplest fractional differential equation governing relaxation processes. Through the sections of the text we plan to address the reader in this pathway towards the main applications of the Mittag-Leffler function that has induced us in the past to define it as the *Queen Function of the Fractional Calculus*. These applications concern some noteworthy stochastic processes and the time fractional diffusion-wave equation We expect that in the next future this function will gain more credit in the science of complex systems. Finally, in an appendix we sketch some historical aspects related to the author’s acquaintance with this function.

## 1. Introduction

For a few decades, the special transcendental function known as the Mittag-Leffler function has attracted the increasing attention of researchers because of its key role in treating problems related to integral and differential equations of fractional order.

Since its introduction in 1903–1905 by the Swedish mathematician Mittag-Leffler at the beginning of the last century up to the 1990s, this function was seldom considered by mathematicians and applied scientists.

Before the 1990s, from a mathematical point of view, we recall the 1930 paper by Hille and Tamarkin [1] on the solutions of the Abel integral equation of the second kind, and the books by Davis [2], Sansone & Gerretsen [3], Dzherbashyan [4] (unfortunately in Russian), and finally Samko et al. [5]. Particular mention would be for the 1955 Handbook of High Transcendental Functions of the Bateman project [6], where this function was treated in Volume 3, in the chapter devoted to miscellaneous functions. For former applications we recall an interesting note by Davis [2] reporting previous research by Dr. Kenneth S. Cole in connection with nerve conduction, and the papers by Cole & Cole [7], Gross [8] and Caputo & Mainardi [9,10], where the Mittag-Leffler function was adopted to represent the responses in dielectric and viscoelastic media. More information are found in the Appendix of this survey.

In the 1960’s the Mittag-Leffler function started to exit from the realm of miscellaneous functions because it was considered as a special case of the general class of Fox *H* functions, that can exhibit an arbitrary number of parameters in their integral Mellin-Barnes representation. However, in our opinion, this classification in a too general framework has, to some extent, obscured the relevance and the applicability of this function in applied sciences. In fact, most mathematical models are based on a small number of parameters, say 1 or 2 or 3, so that a general theory may be confusing whereas the adoption of a generalized Mittag-Leffler function with 2 or 3 indices may be sufficient.

Nowadays it is well recognized that the Mittag-Leffler function plays a fundamental role in Fractional Calculus even if with a single parameter (as originally introduced by Mittag-Leffler) just to be worth of being referred to as the *Queen Function of Fractional Calculus*, see Mainardi & Gorenflo [11]. We find some information on the Mittag-Leffler functions in any treatise on Fractional Calculus but for more details we refer the reader to the surveys of Haubold, Mathai and Saxena [12] and by Van Mieghem [13] and to the treatise by Gorenflo et al. [14], just devoted to Mittag-Leffler functions, related topics and applications.

The plan of this survey is the following. We start to give in Section 2 the main definitions and properties of the Mittag-Leffler function in one parameter with related Laplace transforms. Then in Section 3 we describe its use in the simplest fractional relaxation equation pointing out its compete monotonicity. The asymptotic properties are briefly discussed in Section 4. In Section 5 we briefly discuss the so called generalized Mittag-Leffler function, that is the 2-parameter Mittag-Leffler function. Of course further generalization to 3 and more parameter will be referred to specialized papers and books. Then in the following sections we discuss the application of the Mittag-Leffler function in some noteworthy stochastic processes. We start in Section 6 with the fractional Poisson process, and then in Section 7 with its application of the thinning of renewal processes. The main application are dealt in Section 8 where we discuss the continuous time random walks (CTRW) and then in Section 9 we point out the asymptotic universality. In Section 10 we discuss the time fractional diffusion-wave processes pointing out the role of the Mittag-Leffler functions in two parameters and their connection with the basic Wright functions. In Appendix A we find worthwhile to report the acquaintance of the author with the Mittag-Leffler functions started in the late 1960s and continued up to nowadays.

We recall that Section 3, Section 4, Section 5, Section 6, Section 7, Section 8, Section 9 and Section 10 are taken from several papers by the author, published alone and with colleagues and former students. Furthermore we have not considered other applications of the Mittag-Leffler functions including, for example, anomalous diffusion theory in terms of fractional and generalized Langevin equations. On this respect we refer the readers to the articles of the author, see References [15,16], and to the recent book by Sandev and Tomovski [17] and references therein. For many items related to the Mittag-Leffler functions we refer again to the treatise by Gorenflo et al. [14].

## 2. The Mittag-Leffler Functions: Definitions and Laplace Transforms

The Mittag-Leffler function is defined by the following power series, convergent in the whole complex plane,

(1) $$E_{\alpha }\left(z\right):=\sum_{n=0}^{\infty }\frac{z^{n}}{\Gamma (\alpha n+1)}\;,\;\alpha >0\;,\;z\in ｜\;C\;.$$

We recognize that it is an entire function of order 1/α providing a simple generalization of the exponential function exp(z) to which it reduces for α=1. For detailed information on the Mittag-Leffler-type functions and their Laplace transforms the reader may consult e.g., [6,18,19] and the recent treatise by Gorenflo et al. [14].

We also note that for the convergence of the power series in (1) the parameter α may be complex provided that ℜ(α)>0. The most interesting properties of the Mittag-Leffler function are associated with its asymptotic expansions as z→∞ in various sectors of the complex plane.

In this paper we mainly consider the Mittag-Leffler function of order α∈(0,1) on the negative real semi-axis where is known to be completely monotone (CM) due a classical result by Pollard [20], see also Feller [21].

Let us recall that a function ϕ(t) with $t\in {I\;R}^{+}$ is called a completely monotone (CM) function if it is non-negative, of class $C^{\infty }$, and ${\left(-1\right)}^{n}\phi^{\left(n\right)}\left(t\right)\geq 0$ for all n∈IN. Then a function ψ(t) with $t\in {I\;R}^{+}$ is called a Bernstein function if it is non-negative, of class $C^{\infty }$, with a CM first derivative. These functions play fundamental roles in linear hereditary mechanics to represent relaxation and creep processes, see, for example, Mainardi [22]. For mathematical details we refer the interested reader to the survey paper by Miller and Samko [23] and to the treatise by Schilling et al. [24].

In particular we are interested in the function

(2) $$e_{\alpha }\left(t\right):=E_{\alpha }\left(-t^{\alpha }\right)=\sum_{n=0}^{\infty }{\left(-1\right)}^{n}\frac{t^{\alpha n}}{\Gamma (\alpha n+1)}\;,\;t>0\;,\;0<\alpha \leq 1\;,$$

whose Laplace transform pair reads

(3) $$e_{\alpha }\left(t\right)\;÷\;\frac{s^{\alpha -1}}{s^{\alpha }+1}\;,\;\alpha >0\;.$$

Here we have used the notation ÷ to denote the juxtaposition of a function of time f(t) with its Laplace transform

$$\tilde{f}\left(s\right)=\int_{0}^{\infty }e^{-st}\;f\left(t\right)\;dt\;.$$

The pair (3) can be proved by transforming term by term the power series representation of $e_{\alpha }\left(t\right)$ in the R.H.S of (2). Similarly we can prove the following Laplace transform pair for its time derivative

(4) $$e_{\alpha }^{\prime }\left(t\right)=\frac{d}{dt}E_{\alpha }\left(-t^{\alpha }\right)\;÷\;-\frac{1}{s^{\alpha }+1}\;,\;\alpha >0\;.$$

For this Laplace transform pair we can simply apply the usual rule for the Laplace transform for the first derivative of a function, that reads

$$\frac{d}{dt}f\left(t\right)\;÷\;s\;\tilde{f}\left(s\right)-f\left(0^{+}\right)\;.$$

## 3. The Mittag-Leffler Function in Fractional Relaxation Processes

For readers’ convenience let us briefly outline the topic concerning the generalization via fractional calculus of the first-order differential equation governing the phenomenon of (exponential) relaxation. Recalling (in non-dimensional units) the initial value problem

(5) $$\frac{du}{dt}=-u\left(t\right)\;,\;t\geq 0\;,\;\mathrm{with}\;u\left(0^{+}\right)=1\;$$

whose solution is

(6) u(t)=exp(−t),

the following two alternatives with α∈(0,1) are offered in the literature:

(7) $$\left(a\right)\;\frac{du}{dt}=-D_{t}^{1-\alpha }\;u\left(t\right)\;,\;t\geq 0\;,\;\mathrm{with}\;u\left(0^{+}\right)=1\;,$$

(8) $$\left(b\right){\;}_{*}D_{t}^{\alpha }\;u\left(t\right)=-u\left(t\right)\;,\;t\geq 0\;,\;\mathrm{with}\;u\left(0^{+}\right)=1\;,$$

where $D_{t}^{1-\alpha }$ and ${\;}_{*}D_{t}^{\alpha }$ denote the fractional derivative of order 1−α in the Riemann-Liouville sense and the fractional derivative of order α in the Caputo sense, respectively.

For a generic order μ∈(0,1) and for a sufficiently well-behaved function f(t) with $t\in {I\;R}^{+}$ the above derivatives are defined as follows, see for example, Gorenflo and Mainardi [18], Podlubny [19],

(9) $$\left(a\right)\;D_{t}^{\mu }\;f\left(t\right)=\frac{1}{\Gamma (1-\mu )}\;\frac{d}{dt}[\int_{0}^{t}\;\frac{f(\tau )}{{\left(t-\tau \right)}^{\mu }}\;d\tau ]\;,$$

(10) $$\left(b\right){\;}_{*}D_{t}^{\mu }\;f\left(t\right)=\frac{1}{\Gamma (1-\mu )}\int_{0}^{t}\;\frac{f^{\prime }\left(\tau \right)}{{\left(t-\tau \right)}^{\mu }}\;d\tau \;.$$

Between the two derivatives we have the relationship

(11) $${}_{*}D_{t}^{\mu }\;f\left(t\right)=D_{t}^{\mu }\;f\left(t\right)-f\left(0^{+}\right)\;\frac{t^{-\mu }}{\Gamma (1-\mu )}=D_{t}^{\mu }\;[f\left(t\right)-f\left(0^{+}\right)]\;.$$

Both derivatives in the limit $\mu \to 1^{-}$ reduce to the standard first derivative but for $\mu \to 0^{+}$ we have

(12) $$D_{t}^{\mu }f\left(t\right)\to f\left(t\right)\;,{\;}_{*}D_{t}^{\mu }f\left(t\right)=f\left(t\right)-f\left(0^{+}\right)\;,\;\mu \to 0^{+}\;.$$

In analogy to the standard problem (5), we solve the problems (7) and (8) with the Laplace transform technique, using the rules pertinent to the corresponding fractional derivatives, that we recall hereafter for a generic order μ∈(0,1),

(13) $$\left(a\right)\;D_{t}^{\mu }\;f\left(t\right)\;÷s^{\mu }\tilde{f}\left(s\right)-g\left(0^{+}\right)\;,\;g\left(0^{+}\right)=\frac{1}{\Gamma (1-\mu )}\;\lim_{t\to 0^{+}}\int_{0}^{t}{\left(t-\tau \right)}^{-\mu }\;f\left(\tau \right)\;d\tau \;.$$

(14) $$\left(b\right){\;}_{*}D_{t}^{\mu }\;f\left(t\right)\;÷s^{\mu }\tilde{f}\left(s\right)-f\left(0^{+}\right)\;.$$

We note that it is generally more cumbersome to use the Laplace transform pair for the Rieaman Liouville derivative (13) that for the Capute derivative (14). Indeed the rule (13) requires the initial value of the fractional integral of f(t) whereas the rule (14) simply requires the initial value of f(t). For this property the Caputo derivative is mostly used in physical problems where finite initial values are given.

Then we recognize that the problems (a) and (b) are equivalent since the Laplace transform of the solution in both cases comes out as

(15) $$\tilde{u}\left(s\right)=\frac{s^{\alpha -1}}{s^{\alpha }+1}\;,$$

that yields, in virtue of the Laplace transform pair (3)

(16) $$u\left(t\right)=e_{\alpha }\left(t\right):=E_{\alpha }\left(-t^{\alpha }\right)\;.$$

We thus recognize that the Mittag-Leffler function provides the solution to the fractional relaxation equation, as outlined, for example, by Gorenflo and Mainardi [18], Mainardi and Gorenflo [11], and Mainardi [22].

Furthermore, by anti-transforming the R.H.S of (3) by using the complex Bromwich formula, and taking into account for 0<α<1 the contribution from branch cut on the negative real semi-axis (the denominator $s^{\alpha }+1$ does nowhere vanish in the cut plane −π≤args≤π), we get, see the survey by Gorenflo and Mainardi [18],

(17) $$e_{\alpha }\left(t\right)=\int_{0}^{\infty }\;\;e^{-rt}K_{\alpha }\left(r\right)\;dr\;,$$

where

(18) Kα(r)=∓1πIm{sα−1sα+1|s=re±iπ}=1πrα−1sin(απ)r2α+2rαcos(απ)+1≥0.

We note that this formula was obtained as a simple exercise of complex analysis without be aware of the Titchmarsh formula for inversion of Laplace transforms [25], revised by Gross and Levi [26] and by Gross [27]. This formula is rarely outlined in books on Laplace transforms so we refer the reader for example to Apelblat’s book [28] for its presence.

Since $K_{\alpha }\left(r\right)$ is non-negative for all *r* in the integral, the above formula proves that $e_{\alpha }\left(t\right)$ is a CM function in view of the Bernstein theorem. This theorem provides a necessary and sufficient condition for a CM function as a real Laplace transform of a non-negative measure.

However, the CM property of $e_{\alpha }\left(t\right)$ can also be seen as a consequence of the result by Pollard [20] because the transformation $x=t^{\alpha }$ is a Bernstein function for α∈(0,1). In fact it is known that a CM function can be obtained by composing a CM with a Bernstein function based on the following theorem: *Let ϕ(t) be a CM function and let ψ(t) be a Bernstein function, then ϕ[ψ(t)] is a CM function.*

As a matter of fact, $K_{\alpha }\left(r\right)$ provides an interesting spectral representation of $e_{\alpha }\left(t\right)$ in frequencies. With the change of variable τ=1/r we get the corresponding spectral representation in relaxation times, namely

(19) $$e_{\alpha }\left(t\right)=\int_{0}^{\infty }e^{-t/\tau }H_{\alpha }\left(\tau \right)\;d\tau \;,\;H_{\alpha }\left(\tau \right)=\tau^{-2}\;K_{\alpha }\left(1/\tau \right)\;,$$

that can be interpreted as a continuous distributions of elementary (i.e., exponential) relaxation processes. As a consequence we get the identity between the two spectral distributions, that is

(20) $$K_{\alpha }\left(r\right)=H_{\alpha }\left(\tau \right)=\frac{1}{\pi }\;\frac{\tau^{\alpha -1}\;\sin\;\left(\alpha \pi \right)}{\tau^{2\alpha }+2\;\tau^{\alpha }\;\cos\;\left(\alpha \pi \right)+1}\;,$$

a surprising fact pointed out in Linear Viscoelasticity by the author in his book [22]. This kind of universal/scaling property seems a peculiar one for our Mittag-Leffler function $e_{\alpha }\left(t\right)$.

**Figure 1:**
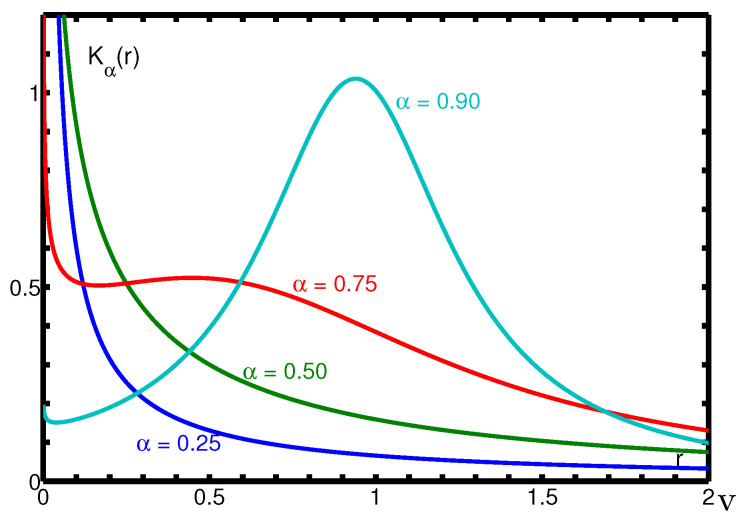
The spectral function $K_{\alpha }\left(r\right)$ for α=0.25,0.50,0.75,0.90 in the frequency range 0≤r≤2.

In Figure 1, we show $K_{\alpha }\left(r\right)$ for some values of the parameter α. Of course for α=1 the Mittag-Leffler function reduces to the exponential function exp(−t) and the corresponding spectral distribution is the Dirac delta generalized function centred at r=1, namely δ(r−1).

**Figure 2:**
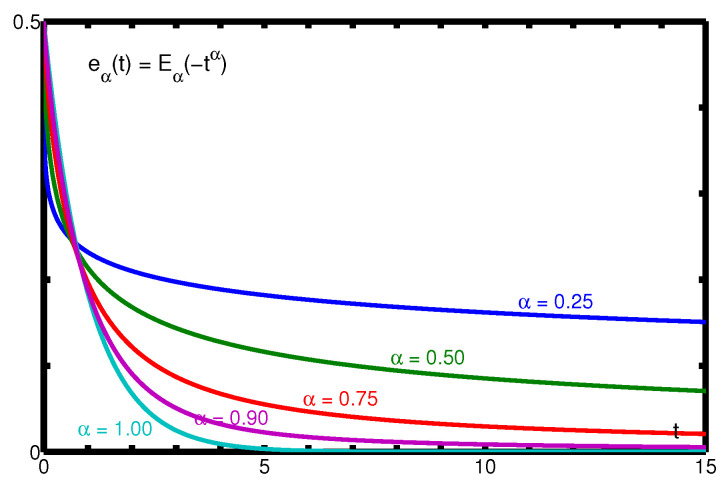
The Mittag-Leffler function $e_{\alpha }\left(t\right)$ for α=0.25,0.50,0.75,0.90,1. in the time range 0≤t≤15.

In Figure 2, we show some plots of $e_{\alpha }\left(t\right)$ for some values of the parameter α. It is worth to note the different rates of decay of $e_{\alpha }\left(t\right)$ for small and large times. In fact the decay is very fast as $t\to 0^{+}$ and very slow as t→+∞.

The Mittag-Leffler function turns out the basic function in relaxation processes of physical interested as occurring in viscoelastic and dielectric materials. We refer the readers for viscoelasticity, that is, to the contribution of the author including References [22,29,30] whereas for dielectric materials to the survey by Garrappa et al. [31]. For the pioneers who have pointed out the role of the Mittagf-Leffler function in mechanical and dielectric relaxation processes we refer to the recent survey by Mainardi and Consiglio [32].

## 4. Asymptotic Approximations to the Mittag-Lefler Function

We now report the two common asymptotic approximations of our Mittag-Lefflrer function. Indeed, it is common to point out that the function $e_{\alpha }\left(t\right)$ matches for $t\to 0^{+}$ with a stretched exponential with an infinite negative derivative, whereas as t→∞ with a negative power law. The short time approximation is derived from the convergent power series representation (2). In fact,

(21) $$e_{\alpha }\left(t\right)=1-\frac{t^{\alpha }}{\Gamma (1+\alpha )}+\cdots ∼\exp\left[-\frac{t^{\alpha }}{\Gamma (1+\alpha )}\right]\;,\;t\to 0\;.$$

The long time approximation is derived from the asymptotic power series representation of $e_{\alpha }\left(t\right)$ that turns out to be, see [6]

(22) $$e_{\alpha }\left(t\right)∼\sum_{n=1}^{\infty }{\left(-1\right)}^{n-1}\;\frac{t^{-\alpha n}}{\Gamma (1-\alpha n)}\;,\;t\to \infty \;,$$

so that, at the first order,

(23) $$e_{\alpha }\left(t\right)∼\frac{t^{-\alpha }}{\Gamma (1-\alpha )}\;,\;t\to \infty \;.$$

As a consequence the function $e_{\alpha }\left(t\right)$ interpolates for intermediate time *t* between the stretched exponential and the negative power law. The stretched exponential models the very fast decay for small time *t*, whereas the asymptotic power law is due to the very slow decay for large time *t*. In fact, we have the two commonly stated asymptotic representations:

(24) $$e_{\alpha }\left(t\right)∼\left\{\begin{array}{cc} e_{\alpha }^{0}\left(t\right):=\exp\left[-\frac{t^{\alpha }}{\Gamma (1+\alpha )}\right]\;, & t\to 0\;;\\ e_{\alpha }^{\infty }\left(t\right):=\frac{t^{-\alpha }}{\Gamma (1-\alpha )}=\frac{\sin(\alpha \pi )}{\pi }\;\frac{\Gamma (\alpha )}{t^{\alpha }}\;, & t\to \infty \;. \end{array}\right.$$

The stretched exponential replaces the rapidly decreasing expression $1-t^{\alpha }/\Gamma (1+\alpha )$ from (21). Of course, *for sufficiently small and for sufficiently large values of t* we have the inequality

(25) $$e_{\alpha }^{0}\left(t\right)\leq e_{\alpha }^{\infty }\left(t\right)\;,\;0<\alpha <1\;.$$

**Figure 3:**
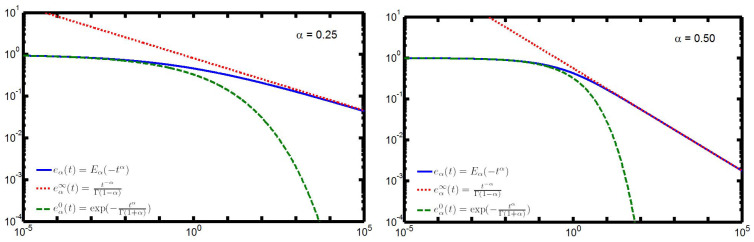
Approximations $e_{\alpha }^{0}\left(t\right)$ (dashed line) and $e_{\alpha }^{\infty }\left(t\right)$ (dotted line) to $e_{\alpha }\left(t\right)$ in $10^{-5}\leq t\leq 10^{+5}$ for α=0.25 (LEFT) and for α=0/50 (RIGHT).

**Figure 4:**
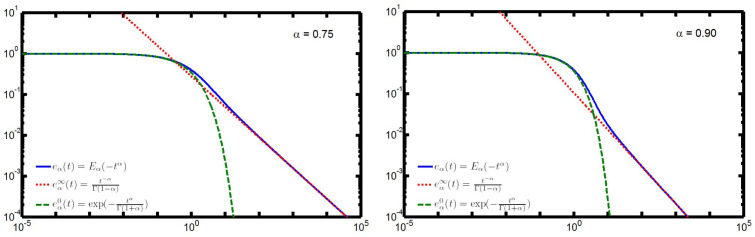
Approximations $e_{\alpha }^{0}\left(t\right)$ (dashed line) and $e_{\alpha }^{\infty }\left(t\right)$ (dotted line) to $e_{\alpha }\left(t\right)$ (LEFT) and the corresponding relative errors (RIGHT) in $10^{-5}\leq t\leq 10^{+5}$ for α=0/75 (LEFT) and for α=0.90 (RIGHT).

In Figure 3 and Figure 4, we compare for α=0.25,0.5,0.75,0.90 in logarithmic scales the function $e_{\alpha }\left(t\right)$ (continuous line) and its asymptotic representations, the stretched exponential $e_{\alpha }^{0}\left(t\right)$ valid for t→0 (dashed line) and the power law $e_{\alpha }^{\infty }\left(t\right)$ valid for t→∞ (dotted line). We have chosen the time range $10^{-5}\leq t\leq 10^{+5}$.

We note from Figure 3 and Figure 4 that, whereas the plots of $e_{\alpha }^{0}\left(t\right)$ remain always under the corresponding ones of $e_{\alpha }\left(t\right)$, the plots of $e_{\alpha }^{\infty }\left(t\right)$ start above those of $e_{\alpha }\left(t\right)$ but, at a certain point, an intersection may occur so changing the sign of the relative errors. The interested reader may consul the plots of the relative errors in the 2014 paper by the author [33] from which, in particular, Figure 1, Figure 2, Figure 3 and Figure 4 have been extracted.

## 5. The Generalized Mittag-Leffler Function

In this survey we will devote our attention mainly to the classical Mittag-Leffler in one parameter α as introduced by Mittag-Leffler since 1903 defined by the power series in (1). We have just learned from the instructive E-print by Van Mieghem [13] that the series (1) was discussed by Hadamard in 1893, that is 10 years earlier than Mittag-Leffler himself.

As a matter of fact a straightforward generalization of the classical Mittag-Leffler function is obtained by replacing the additive constant 1 in the argument of the Gamma function in (1) by an arbitrary complex parameter β. It was formerly considered in 1905 by Reference [34] and soon later by Mittag-leffler himself, almost incidentally in one of his notes. Later, in the 1950’s, such generalization was investigated by Humbert and Agarwal, with respect to the Laplace transformation, see References [35,36,37]. Usually, when dealing with Laplace transform pairs, the parameter β is required to be real and positive like α.

For this function we agree to use the notation

(26) $$E_{\alpha ,\beta }\left(z\right):=\sum_{n=0}^{\infty }\frac{z^{n}}{\Gamma (\alpha n+\beta )}\;,\;ℜ\alpha >0\;,\;\;\beta \in ｜\;C\;,\;z\in ｜\;C\;.$$

Of course $E_{\alpha ,1}\left(z\right)\equiv E_{\alpha }\left(z\right)$. The series is still convergent for all the complex plane ｜C so the function (26) is still entire for ℜ(α)>0 for any β∈｜C with order 1/ℜα so the additional parameter play any role on this respect. However the Laplace transform pairs concerning the Mittag-Leffler function (26) and its derivative are known to be with α,β>0 and ${ℜ\left(s\right)>|\lambda |}^{1\alpha }$, see, for example, Refs. [14,19,22],

(27) $$t^{\beta -1}\;E_{\alpha ,\beta }\left(-\lambda \;t^{\alpha }\right)\;÷\;\frac{s^{\alpha -\beta }}{s^{\alpha }+\lambda }=\frac{s^{-\beta }}{1+\lambda s^{-\alpha }}\;.$$

and

(28) $$t^{\alpha k+\beta -1}\;E_{\alpha ,\beta }^{\left(k\right)}\left(\lambda t^{\alpha }\right)\;÷\;\frac{k!\;s^{\alpha -\beta }}{{\left(s^{\alpha }-\lambda \right)}^{k+1}}\;,\;k=0,1,2,\ldots \;.$$

We also note the following relation concerning the first derivative of the classical Mittag-Leffler function with the two-parameter Mittag-Leffler function usually overlooked by several authors but of easy proof:

(29) $$\phi_{\alpha }\left(t\right):=t^{-(1-\alpha )}\;E_{\alpha ,\alpha }\left(-t^{\alpha }\right)=-\frac{d}{dt}E_{\alpha }\left(-t^{\alpha }\right)\;,\;t\geq 0,\;0<\alpha <1\;.$$

**Figure 5:**
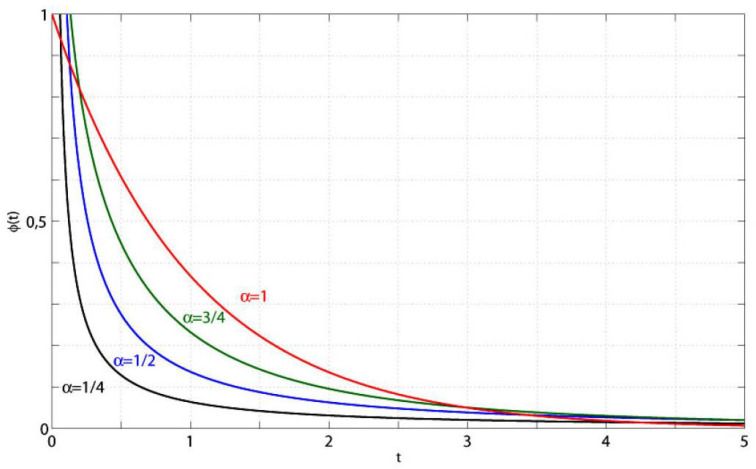
Plots of $\phi_{\alpha }\left(t\right)$ with α=1/4,1/2,3/4,1 versus *t*; for 0≤t≤5.

We report the plot of the function $\phi_{\alpha }\left(t\right)$ herewith in Figure 5.

We note that Mittag-Leffler functions with more than two parameters were also dealt by several authors as pointed out in [14]. In particular, for the 3-parameter Mittag-Leffler function (known as Prabhakar function) and related operators we refer the reader to the recent survey by Giusti et al. [38] and references therein. Kiryakova has dealt in a number of papers the multi-index Mittag-Leffler functions, see for example [39].

## 6. The Fractional Poisson Process and the Mittag-Leffler Function

Hereafter we describe how the Mittag-Leffler function enters in the so-called fractional Poisson process. We are following the original approach by Mainardi et al. in [40] where the fractional Poisson process is referred to as the renewal process of the Mittag-Leffler type. However, an independent approach to the fractional Poisson process was given for example, by Laskin in [41].

### 6.1. Essentials of Renewal Theory

The concept of *renewal process* has been developed as a stochastic model for describing the class of counting processes for which the times between successive events are independent identically distributed (iid) non-negative random variables, obeying a given probability law. These times are referred to as waiting times or inter-arrival times. For more details see, for example, the classical treatises by Cox [42], Feller [21].

For a renewal process having waiting times $T_{1},T_{2},\ldots$, let

(30) $$t_{0}=0\;,\;t_{k}=\sum_{j=1}^{k}T_{j}\;,\;k\geq 1\;.$$

That is $t_{1}=T_{1}$ is the time of the first renewal, $t_{2}=T_{1}+T_{2}$ is the time of the second renewal and so on. In general $t_{k}$ denotes the *k*th renewal.

The process is specified if we know the probability law for the waiting times. In this respect we introduce the *probability density function* (pdf) ϕ(t) and the (cumulative) distribution function Φ(t) so defined:

(31) $$\phi \left(t\right):=\frac{d}{dt}\Phi \left(t\right)\;,\;\Phi \left(t\right):=P\left(T\leq t\right)=\int_{0}^{t}\phi \left(t^{\prime }\right)\;dt^{\prime }\;.$$

When the non-negative random variable represents the lifetime of technical systems, it is common to refer to Φ(t) as to the *failure probability* and to

(32) $$\Psi \left(t\right):=P\left(T>t\right)=\int_{t}^{\infty }\phi \left(t^{\prime }\right)\;dt^{\prime }=1-\Phi \left(t\right)\;,$$

as to the *survival probability*, because Φ(t) and Ψ(t) are the respective probabilities that the system does or does not fail in (0,T]. A relevant quantity is the *counting function* N(t) defined as

(33) $$N\left(t\right):=\max\left\{k|t_{k}\leq t,\;k=0,1,2,\ldots \right\}\;,$$

that represents the effective number of events before or at instant *t*. In particular we have $\Psi \left(t\right)=P\left(N(t)=0\right)\;.$ Continuing in the general theory we set $F_{1}\left(t\right)=\Phi \left(t\right)$, $f_{1}\left(t\right)=\phi \left(t\right)$, and in general

(34) $$F_{k}\left(t\right):=P\left(t_{k}=T_{1}+\cdots +T_{k}\leq t\right)\;,\;f_{k}\left(t\right)=\frac{d}{dt}F_{k}\left(t\right)\;,\;k\geq 1\;,$$

thus $F_{k}\left(t\right)$ represents the probability that the sum of the first *k* waiting times is less or equal *t* and $f_{k}\left(t\right)$ its density. Then, for any fixed k≥1 the normalization condition for $F_{k}\left(t\right)$ is fulfilled because

(35) $$\lim_{t\to \infty }F_{k}\left(t\right)=P\left(t_{k}=T_{1}+\cdots +T_{k}<\infty \right)=1\;.$$

In fact, the sum of *k* random variables each of which is finite with probability 1 is finite with probability 1 itself. By setting for consistency $F_{0}\left(t\right)\equiv 1$ and $f_{0}\left(t\right)=\delta \left(t\right)$, where for the Dirac delta generalized function in ${I\;R}^{+}$ we assume the *formal representation*

$$\delta \left(t\right):=\frac{t^{-1}}{\Gamma (0)}\;,\;t\geq 0\;,$$

we also note that for k≥0 we have

(36) $$P\left(N(t)=k\right):=P\left(t_{k}\leq t\;,\;t_{k+1}>t\right)=\int_{0}^{t}f_{k}\left(t^{\prime }\right)\;\Psi \left(t-t^{\prime }\right)\;dt^{\prime }\;.$$

We now find it convenient to introduce the simplified ∗ notation for the Laplace convolution between two causal well-behaved (generalized) functions f(t) and g(t)

$$\int_{0}^{t}f\left(t^{\prime }\right)\;g\left(t-t^{\prime }\right)\;dt^{\prime }=\left(f\;*\;g\right)\left(t\right)=\left(g\;*\;f\right)\left(t\right)=\int_{0}^{t}f\left(t-t^{\prime }\right)\;g\left(t^{\prime }\right)\;dt^{\prime }\;.$$

Being $f_{k}\left(t\right)$ the pdf of the sum of the *k* iid random variables $T_{1},\ldots ,T_{k}$ with pdfϕ(t), we easily recognize that $f_{k}\left(t\right)$ turns out to be the *k*-fold convolution of ϕ(t) with itself,

(37) $$f_{k}\left(t\right)=\left(\phi^{*k}\right)\left(t\right)\;,$$

so Equation (36) simply reads:

(38) $$P\left(N(t)=k\right)=\left(\phi^{*k}\;*\;\Psi \right)\left(t\right)\;.$$

Because of the presence of Laplace convolutions a renewal process is suited for the Laplace transform method. Throughout this paper we will denote by $\tilde{f}\left(s\right)$ the Laplace transform of a sufficiently well-behaved (generalized) function f(t) according to

$$L\left\{f(t);s\right\}=\tilde{f}\left(s\right)=\int_{0}^{+\infty }e^{\;-st}\;f\left(t\right)\;dt\;,\;s>s_{0}\;,$$

and for δ(t) consistently we will have $\tilde{\delta }\left(s\right)\equiv 1\;.$ Note that for our purposes we agree to take *s* real. We recognize that (38) reads in the Laplace domain

(39) $$L\left\{P\left(N(t)=k\right);s\right\}={\left[\tilde{\phi }\left(s\right)\right]}^{k}\;\tilde{\Psi }\left(s\right)\;,$$

where, using (32),

(40) $$\tilde{\Psi }\left(s\right)=\frac{1-\tilde{\phi }\left(s\right)}{s}\;.$$

### 6.2. The Classical Poisson Process as a Renewal Process

The most celebrated renewal process is the Poisson process characterized by a waiting time pdf of exponential type,

(41) $$\phi \left(t\right)=\lambda \;e^{-\lambda t}\;,\;\lambda >0\;,\;t\geq 0\;.$$

The process has *no memory*. Its moments turn out to be

(42) $$\langle T\rangle =\frac{1}{\lambda }\;,\;\langle T^{2}\rangle =\frac{1}{\lambda^{2}}\;,\;\ldots \;,\;\langle T^{n}\rangle =\frac{1}{\lambda^{n}}\;,\;\ldots \;,$$

and the *survival probability* is

(43) $$\Psi \left(t\right):=P\left(T>t\right)=e^{-\lambda t}\;,\;t\geq 0\;.$$

We know that the probability that *k* events occur in the interval of length *t* is

(44) $$P\left(N(t)=k\right)=\frac{{\left(\lambda t\right)}^{k}}{k!}\;e^{-\lambda t}\;,\;t\geq 0\;,\;k=0,1,2,\ldots \;.$$

The probability distribution related to the sum of *k* iid exponential random variables is known to be the so-called *Erlang distribution* (of order *k*). The corresponding density (the *Erlang* pdf) is thus

(45) $$f_{k}\left(t\right)=\lambda \;\frac{{\left(\lambda t\right)}^{k-1}}{(k-1)!}\;e^{-\lambda t}\;,\;t\geq 0\;,\;k=1,2,\ldots \;,$$

so that the Erlang distribution function of order *k* turns out to be

(46) $$F_{k}\left(t\right)=\int_{0}^{t}f_{k}\left(t^{\prime }\right)\;dt^{\prime }=1-\sum_{n=0}^{k-1}\frac{{\left(\lambda t\right)}^{n}}{n!}\;e^{-\lambda t}=\sum_{n=k}^{\infty }\frac{{\left(\lambda t\right)}^{n}}{n!}\;e^{-\lambda t}\;,\;t\geq 0\;.$$

In the limiting case k=0 we recover $f_{0}\left(t\right)=\delta \left(t\right),\;F_{0}\left(t\right)\equiv 1,\;t\geq 0$.

The results (44)–(46) can easily obtained by using the technique of the Laplace transform sketched in the previous section noting that for the Poisson process we have:

(47) $$\tilde{\phi }\left(s\right)=\frac{\lambda }{\lambda +s}\;,\;\tilde{\Psi }\left(s\right)=\frac{1}{\lambda +s}\;,$$

and for the Erlang distribution:

(48) $${\tilde{f}}_{k}\left(s\right)={\left[\tilde{\phi }\left(s\right)\right]}^{k}=\frac{\lambda^{k}}{{\left(\lambda +s\right)}^{k}}\;,\;{\tilde{F}}_{k}\left(s\right)=\frac{{\left[\tilde{\phi }\left(s\right)\right]}^{k}}{s}=\frac{\lambda^{k}}{s{\left(\lambda +s\right)}^{k}}\;.$$

We also recall that the survival probability for the Poisson renewal process obeys the ordinary differential equation (of relaxation type)

(49) $$\frac{d}{dt}\Psi \left(t\right)=-\lambda \Psi \left(t\right)\;,\;t\geq 0\;;\;\Psi \left(0^{+}\right)=1\;.$$

### 6.3. The Renewal Process of Mittag-Leffler Type

A “fractional” generalization of the Poisson renewal process is simply obtained by generalizing the differential Equation (49) replacing there the first derivative with the integro-differential operator ${\;}_{*}D_{t}^{\beta }$ that is interpreted as the fractional derivative of order β in Caputo’s sense, see Section 2. We write, taking for simplicity λ=1,

(50) $${\;}_{*}D_{t}^{\beta }\;\Psi \left(t\right)=-\Psi \left(t\right)\;,\;t>0\;,\;0<\beta \leq 1\;;\;\Psi \left(0^{+}\right)=1\;.$$

We also allow the limiting case β=1 where all the results of the previous section (with λ=1) are expected to be recovered.

For our purpose we need to recall the Mittag-Leffler function as the natural “fractional” generalization of the exponential function, that characterizes the Poisson process. We again recall that the Mittag-Leffler function of parameter β is defined in the complex plane by the power series

(51) $$E_{\beta }\left(z\right):=\sum_{n=0}^{\infty }\;\frac{z^{n}}{\Gamma (\beta \;n+1)}\;,\;\beta >0\;,\;z\in ｜\;C\;,$$

as stated in Section 2 where the parameter was denoted by α.

The solution of Equation (50) is known to be, see Section 3

(52) $$\Psi \left(t\right)=E_{\beta }\left(-t^{\beta }\right)\;,\;t\geq 0\;,\;0<\beta \leq 1\;,$$

so

(53) $$\phi \left(t\right):=-\frac{d}{dt}\Psi \left(t\right)=-\frac{d}{dt}E_{\beta }\left(-t^{\beta }\right)\;,\;t\geq 0\;,\;0<\beta \leq 1\;.$$

Then, the corresponding Laplace transforms read

(54) $$\tilde{\Psi }\left(s\right)=\frac{s^{\beta -1}}{1+s^{\beta }}\;,\;\tilde{\phi }\left(s\right)=\frac{1}{1+s^{\beta }}\;,\;0<\beta \leq 1\;.$$

Hereafter, we find it convenient to summarize the most relevant features of the functions Ψ(t) and ϕ(t) when 0<β<1. We begin to quote their series expansions convergent in all of IR suitable for $t\to 0^{+}$ and their asymptotic representations for t→∞,

(55) $$\Psi \left(t\right)=\sum_{n=0}^{\infty }\;{\left(-1\right)}^{n}\frac{t^{\beta n}}{\Gamma (\beta \;n+1)}\;∼\;\frac{\sin\;(\beta \pi )}{\pi }\;\frac{\Gamma (\beta )}{t^{\beta }}\;,$$

and

(56) $$\phi \left(t\right)=\frac{1}{t^{1-\beta }}\;\sum_{n=0}^{\infty }\;{\left(-1\right)}^{n}\frac{t^{\beta n}}{\Gamma (\beta \;n+\beta )}\;∼\;\frac{\sin\;(\beta \pi )}{\pi }\;\frac{\Gamma (\beta +1)}{t^{\beta +1}}\;.$$

In contrast to the Poissonian case β=1, in the case 0<β<1 for large *t* the functions Ψ(t) and ϕ(t) no longer decay exponentially but algebraically. As a consequence of the power-law asymptotics the process turns be no longer Markovian but of long-memory type. However, we recognize that for 0<β<1 both functions Ψ(t), ϕ(t) keep the “completely monotonic” character of the Poissonian case. as can be simply derived from Section 2. We recall that *complete monotonicity* of our functions Ψ(t) and ϕ(t) means

(57) $${\left(-1\right)}^{n}\frac{d^{n}}{dt^{n}}\;\Psi \left(t\right)\geq 0\;,\;{\left(-1\right)}^{n}\frac{d^{n}}{dt^{n}}\;\phi \left(t\right)\geq 0\;,\;n=0,1,2,\ldots \;,\;t\geq 0\;,$$

or equivalently, their representability as real Laplace transforms of non-negative generalized functions (or measures).

For the generalizations of Equations (44)–(46), characteristic of the Poisson and Erlang distributions respectively, we must point out the Laplace transform pair

(58) $$t^{\beta \;k}\;E_{\beta }^{\left(k\right)}\left(-t^{\beta }\right)\;÷\;\frac{k!\;s^{\beta -1}}{{\left(1+s^{\beta }\right)}^{k+1}}\;,\;\beta >0\;,\;k=0,1,2,\ldots \;,$$

with $E_{\beta }^{\left(k\right)}\left(z\right):=\frac{d^{k}}{dz^{k}}E_{\beta }\left(z\right)\;,$ that can be deduced from the book by Podlubny, see Equation (1.80) in Reference [19]. Then, by using the Laplace transform pairs (25) and Equations (52), (53), (58) in Equations (37) and (38), we have the *generalized Poisson distribution*,

(59) $$P\left(N(t)=k\right)=\frac{t^{k\;\beta }}{k!}\;E_{\beta }^{\left(k\right)}\left(-t^{\beta }\right)\;,\;k=0,1,2,\ldots$$

and the *generalized Erlang* pdf’s (of order k≥1),

(60) $$f_{k}\left(t\right)=\beta \;\frac{t^{k\beta -1}}{(k-1)!}\;E_{\beta }^{\left(k\right)}\left(-t^{\beta }\right)\;.$$

The *generalized Erlang distribution functions* turn out to be

(61) $$F_{k}\left(t\right)=\int_{0}^{t}f_{k}\left(t^{\prime }\right)\;dt^{\prime }=1-\sum_{n=0}^{k-1}\frac{t^{n\beta }}{n!}\;E_{\beta }^{\left(n\right)}\left(-t^{\beta }\right)=\sum_{n=k}^{\infty }\frac{t^{n\beta }}{n!}\;E_{\beta }^{\left(n\right)}\left(-t^{\beta }\right)\;.$$

## 7. The Gnedenko-Kovalenko Theory of Thinning and the Mittag-Leffler Function

The *thinning* theory for a renewal process has been considered in detail by Gnedenko and Kovalenko [43] in the first edition of their book on Queue theory of 1968. However, the connection with the Laplace transform of the Mittag-Leffler function outlined at the end of this section in Equations (71) and (72), see also [44] and [45], is surprisingly not present in the second edition of the book by Gnedenko & Kovalenko in 1989.

We must note that other authors, like Szántai [46,47] speak of *rarefaction* in place of thinning.

Let us sketch here the essentials of this theory: in the interest of transparency and easy readability we avoid the possible decoration of the relevant power law by multiplying it with a *slowly varying function*.

Denoting by $t_{n}$, n=1,2,3,… the time instants of events of a renewal process, assuming $0=t_{0}<t_{1}<t_{2}<t_{3}<\ldots$, with i.i.d. waiting times $T_{1}=t_{1}\;,\;T_{k}=t_{k}-t_{k-1}$ for k≥2, (generically denoted by T), *thinning* (or *rarefaction*) means that for each positive index *k* a decision is made: the event happening in the instant $t_{k}$ is deleted with probability *p* or it is maintained with probability q=1−p, 0<q<1. This procedure produces a *thinned* or *rarefied* renewal process with fewer events (very few events if *q* is near zero, the case of particular interest) in a moderate span of time.

To compensate for this loss we change the unit of time so that we still have not very few but still a moderate number of events in a moderate span of time. Such change of the unit of time is equivalent to rescaling the waiting time, multiplying it with a positive factor τ so that we have waiting times $\tau T_{1},\tau T_{2},\tau T_{3},\ldots$, and instants $\tau t_{1},\tau t_{2},\tau t_{3},\ldots$, in the rescaled process. Our intention is, vaguely speaking, to dispose on τ in relation to the rarefaction parameter *q* in such a way that for *q* near zero in some sense the “average” number of events per unit of time remains unchanged. In an asymptotic sense we will make these considerations precise.

Denoting by F(t)=P(T≤t) the probability distribution function of the (original) waiting time *T*, by f(t) its density (f(t) is a generalized function generating a probability measure) so that $F\left(t\right)=\int_{0}^{t}f\left(t^{\prime }\right)\;dt^{\prime }$, and analogously by $F_{k}\left(t\right)$ and $f_{k}$(t) the distribution and density, respectively, of the sum of *k* waiting times, we have recursively

(62) $$f_{k}\left(t\right)=\int_{0}^{t}f_{k-1}\left(t-t^{\prime }\right)\;dF\left(t^{\prime }\right)\;,\;\mathrm{for}\;k\geq 2\;.$$

Observing that after a maintained event the next one of the original process is kept with probability *q* but dropped in favour of the second-next with probability pq and, generally, n−1 events are dropped in favour of the *n*-th-next with probability $p^{n-1}\;q$, we get for the waiting time density of the thinned process the formula

(63) $$g_{q}\left(t\right)=\sum_{n=1}^{\infty }q\;p^{n-1}\;f_{n}\left(t\right)\;.$$

With the modified waiting time τT we have

P(τT≤t)=P(T≤t/τ)=F(t/τ),

hence the density f(t/τ)/τ, and analogously for the density of the sum of *n* waiting times $f_{n}\left(t/\tau \right)/\tau$. The density of the waiting time of the rescaled (and thinned) process now turns out as

(64) $$g_{q,\tau }\left(t\right)=\sum_{n=1}^{\infty }q\;p^{n-1}\;f_{n}\left(t/\tau \right)/\tau \;.$$

In the Laplace domain we have ${\tilde{f}}_{n}\left(s\right)={\left(\tilde{f}\left(s\right)\right)}^{n}\;,$ hence (using p=1−q)

(65) $${\tilde{g}}_{q}\left(s\right)=\sum_{n=1}^{\infty }q\;p^{n-1}\;{\left(\tilde{f}\left(s\right)\right)}^{n}=\frac{q\;\tilde{f}\left(s\right)}{1-\left(1-q\right)\;\tilde{f}\left(s\right)}\;,$$

from which by Laplace inversion we can, in principle, construct the waiting time density of the thinned process. By rescaling we get

(66) $${\tilde{g}}_{q,\tau }\left(s\right)=\sum_{n=1}^{\infty }q\;p^{n-1}\;{\left(\tilde{f}\left(\tau s\right)\right)}^{n}=\frac{q\;\tilde{f}\left(\tau s\right)}{1-\left(1-q\right)\;\tilde{f}\left(\tau s\right)}\;.$$

Being interested in stronger and stronger thinning (*infinite thinning*) let us now consider a scale of processes with the parameters τ (of *rescaling*) and *q* (of *thinning*), with *q* tending to zero *under a scaling relation q=q(τ) yet to be specified*.

We have essentially two cases for the waiting time distribution: its expectation value is finite or infinite. In the first case we put

(67) $$\lambda =\int_{0}^{\infty }t^{\prime }\;f\left(t^{\prime }\right)\;dt^{\prime }<\infty \;.$$

In the second case we assume a queue of power law type (dispensing with a possible decoration by a function slowly varying at infinity)

(68) $$\Psi \left(t\right):=\int_{t}^{\infty }f\left(t^{\prime }\right)\;dt^{\prime }∼\frac{c}{\beta }t^{-\beta }\;,\;t\to \infty \;\mathrm{if}\;0<\beta <1\;.$$

Then, by the Karamata theory (see References [21,48]) the above conditions mean in the Laplace domain

(69) $$\tilde{f}\left(s\right)=1-\lambda \;s^{\beta }+o\left(s^{\beta }\right)\;,\;\mathrm{for}\;s\to 0^{+}\;,$$

with a positive coefficient λ and 0<β≤1. The case β=1 obviously corresponds to the situation with finite first moment (2.6a), whereas the case 0<β<1 is related to a power law queue with c=λΓ(β+1)sin(βπ)/π.

Now, passing to the limit of q→0 of infinite thinning under the scaling relation

(70) $$q=\lambda \;\tau^{\beta }\;,\;0<\beta \leq 1\;,$$

between the positive parameters *q* and τ, the Laplace transform of the rescaled density $\tilde{g_{q,\tau }}\left(s\right)$ in (66) of the thinned process tends for fixed *s* to

(71) $$\tilde{g}\left(s\right)=\frac{1}{1+s^{\beta }}\;,$$

which corresponds to the Mittag-Leffler density

(72) $$g\left(t\right)=-\frac{d}{dt}E_{\beta }\left(-t^{\beta }\right)=\phi^{ML}\left(t\right)\;.$$

Let us remark that Gnedenko and Kovalenko obtained (71) as the Laplace transform of the limiting density but did not identify it as the Laplace transform of a Mittag-Leffler type function. Observe that in the special case λ<∞ we have β=1, hence as the limiting process the Poisson process, as formerly shown in 1956 by Rényi [49].

## 8. The Continuous Time Random Walk (CTRW) and the Mittag-Leffler Function

The name *continuous time random walk* (CTRW) became popular in physics after Montroll and Weiss (just to cite the pioneers) published a celebrated series of papers on random walks for modelling diffusion processes on lattices, see, for example, Reference [50], and the book by Weiss [51] with references therein. CTRWs are rather good and general phenomenological models for diffusion, including processes of anomalous transport, that can be understood in the framework of the classical renewal theory. In fact a CTRW can be considered as a compound renewal process (a simple renewal process with reward) or a random walk *subordinated* to a simple renewal process. Hereafter we will mainly follow the approach by Gorenflo & Mainardi, see, for example, Reference [52].

A spatially one-dimensional CTRW is generated by a sequence of independent identically distributed (iid) positive random waiting times $T_{1},T_{2},T_{3},\ldots ,$ each having the same probability density function ϕ(t),t>0, and a sequence of iid random jumps $X_{1},X_{2},X_{3},\ldots ,$ in IR, each having the same probability density w(x),x∈IR.

Let us remark that, for ease of language, we use the word density also for generalized functions in the sense of Gel’fand & Shilov [53], that can be interpreted as probability measures. Usually the *probability density functions* are abbreviated by pdf. We recall that ϕ(t)≥0 with $\int_{0}^{\infty }\phi \left(t\right)\;dt=1$ and w(x)≥0 with $\int_{-\infty }^{+\infty }w\left(x\right)\;dx=1$.

Setting $t_{0}=0\;,$$\;t_{n}=T_{1}+T_{2}+\ldots T_{n}$ for n∈IN, the wandering particle makes a jump of length $X_{n}$ in instant $t_{n}$, so that its position is $x_{0}=0$ for $0\leq t<T_{1}=t_{1}\;,$ and $x_{n}=X_{1}+X_{2}+\ldots X_{n}\;,$ for $t_{n}\leq t<t_{n+1}\;.$ We require the distribution of the waiting times and that of the jumps to be independent of each other. So, we have a compound renewal process (a renewal process with reward), compare Reference [42].

By natural probabilistic arguments we arrive at the *integral equation* for the probability density p(x,t) (a density with respect to the variable *x*) of the particle being in point *x* at instant t,

(73) $$p\left(x,t\right)=\delta \left(x\right)\;\Psi \left(t\right)\;+\int_{0}^{t}\;\;\phi \left(t-t^{\prime }\right)\;\left[\int_{-\infty }^{+\infty }\;\;w\left(x-x^{\prime }\right)\;p\left(x^{\prime },t^{\prime }\right)\;dx^{\prime }\right]\;dt^{\prime }\;,$$

in which δ(x) denotes the Dirac generalized function, and the *survival function*

(74) $$\Psi \left(t\right)=\int_{t}^{\infty }\phi \left(t^{\prime }\right)\;dt^{\prime }$$

denotes the probability that at instant *t* the particle is still sitting in its starting position x=0. Clearly, Equation (73) satisfies the initial condition $p\left(x,0^{+}\right)=\delta \left(x\right)$.

Note that the *special choice*

(75) w(x)=δ(x−1)

gives the *pure renewal process*, with position x(t)=N(t), denoting the *counting function*, and with jumps all of length 1 in positive direction happening at the renewal instants.

For many purposes the integral Equation (73) of CTRW can be easily treated by using the Laplace and Fourier transforms. Writing these as

$$L\left\{f(t);s\right\}=\tilde{f}\left(s\right):=\int_{0}^{\infty }\;\;e^{\;-st}\;f\left(t\right)\;dt\;,\;F\left\{g(x);\kappa \right\}=\hat{g}\left(\kappa \right):=\int_{-\infty }^{+\infty }\;\;e^{\;+i\kappa x}\;g\left(x\right)\;dx\;,$$

then in the Laplace-Fourier domain Equation (73) reads

(76) $$\hat{\tilde{p}}\left(\kappa ,s\right)=\frac{1-\tilde{\phi }\left(s\right)}{s}+\tilde{\phi }\left(s\right)\;\hat{w}\left(\kappa \right)\;\hat{\tilde{p}}\left(\kappa ,s\right)\;.$$

Introducing formally in the Laplace domain the auxiliary function

(77) $$\tilde{H}\left(s\right)=\frac{1-\tilde{\phi }\left(s\right)}{s\;\tilde{\phi }\left(s\right)}=\frac{\tilde{\Psi }\left(s\right)}{\tilde{\phi }\left(s\right)}\;,\;\mathrm{hence}\;\tilde{\phi }\left(s\right)=\frac{1}{1+s\tilde{H}\left(s\right)}\;,$$

and assuming that its Laplace inverse H(t) exists, we get, following Mainardi et al. [54], in the Laplace-Fourier domain the equation

(78) $$\tilde{H}\left(s\right)\;\left[s\hat{\tilde{p}}\left(\kappa ,s\right)-1\right]=\left[\hat{w}\left(\kappa \right)-1\right]\;\hat{\tilde{p}}\left(\kappa ,s\right)\;,$$

and in the space-time domain the generalized Kolmogorov-Feller equation

(79) $$\int_{0}^{t}H\left(t-t^{\prime }\right)\;\frac{\partial }{\partial t^{\prime }}p\left(x,t^{\prime }\right)\;dt^{\prime }=-p\left(x,t\right)+\int_{-\infty }^{+\infty }w\left(x-x^{\prime }\right)\;p\left(x^{\prime },t\right)\;dx^{\prime },$$

with p(x,0)=δ(x), where H(t) acts as a *memory function*.

If the Laplace inverse H(t) of the formally introduced function $\tilde{H}\left(s\right)$ does not exist, we can formally set $\tilde{K}\left(s\right)=1/\tilde{H}\left(s\right)$ and multiply (78) with $\tilde{K}\left(s\right)$. Then, if K(t) exists, we get in place of (79) the alternative form of the generalized Kolmogorov-Feller equation

(80) $$\frac{\partial }{\partial t}p\left(x,t\right)\;=\int_{0}^{t}K\left(t-t^{\prime }\right)\;\left[-p\left(x,t^{\prime }\right)+\int_{-\infty }^{+\infty }w\left(x-x^{\prime }\right)\;p\left(x^{\prime },t^{\prime }\right)\;dx^{\prime }\right]\;dt^{\prime }\;,$$

with p(x,0)=δ(x). where K(t) acts as a *memory function*.

Special choices of the memory function H(t) are (i) and (ii), see Equations (81) and (85):

(81) $$\left(i\right)\;H\left(t\right)=\delta \left(t\right)\;\mathrm{corresponding}\;\mathrm{to}\;\tilde{H}\left(s\right)=1\;,$$

giving the *exponential waiting time* with

(82) $$\tilde{\phi }\left(s\right)=\frac{1}{1+s},\;\phi \left(t\right)=\Psi \left(t\right)=e^{\;-t}\;.$$

In this case we obtain in the Fourier- Laplace domain

(83) $$s\hat{\tilde{p}}\left(\kappa ,s\right)-1=\left[\hat{w}\left(\kappa \right)-1\right]\;\hat{\tilde{p}}\left(\kappa ,s\right)\;,$$

and in the space-time domain the *classical Kolmogorov-Feller equation*

(84) $$\frac{\partial }{\partial t}p\left(x,t\right)=-p\left(x,t\right)+\int_{-\infty }^{+\infty }w\left(x-x^{\prime }\right)\;p\left(x^{\prime },t\right)\;dx^{\prime }\;,\;p\left(x,0\right)=\delta \left(x\right)\;.$$

(85) $$\left(\mathrm{ii}\right)\;H\left(t\right)=\frac{t^{-\beta }}{\Gamma (1-\beta )}\;,\;0<\beta <1\;,\;\mathrm{corresponding}\;\mathrm{to}\;\tilde{H}\left(s\right)=s^{\beta -1}\;,$$

giving the *Mittag-Leffler waiting time* with

(86) $$\tilde{\phi }\left(s\right)=\frac{1}{1+s^{\beta }},\;\phi \left(t\right)=-\frac{d}{dt}E_{\beta }\left(-t^{\beta }\right)=\phi^{ML}\left(t\right),\;\Psi \left(t\right)=E_{\beta }\left(-t^{\beta }\right)\;.$$

In this case we obtain in the Fourier-Laplace domain

(87) $$s^{\beta -1}\;\left[s\hat{\tilde{p}}\left(\kappa ,s\right)-1\right]=\left[\hat{w}\left(\kappa \right)-1\right]\;\hat{\tilde{p}}\left(\kappa ,s\right)\;,$$

and in the space-time domain the *time fractional Kolmogorov-Feller equation*

(88) $${\;}_{*}D_{t}^{\beta }\;p\left(x,t\right)=-p\left(x,t\right)+\int_{-\infty }^{+\infty }w\left(x-x^{\prime }\right)\;p\left(x^{\prime },t\right)\;dx^{\prime }\;,\;p\left(x,0^{+}\right)=\delta \left(x\right)\;,$$

where ${\;}_{*}D_{t}^{\beta }\;$ denotes the fractional derivative of of order β in the Caputo sense, see Section 3.

The time fractional Kolmogorov-Feller equation can be also expressed via the Riemann-Liouville fractional derivative $\;D_{t}^{1-\beta }$, see again Section 3, that is

(89) $$\frac{\partial }{\partial t}p\left(x,t\right)=\;D_{t}^{1-\beta }\;\left[-p\left(x,t\right)+\int_{-\infty }^{+\infty }w\left(x-x^{\prime }\right)\;p\left(x^{\prime },t\right)\;dx^{\prime }\right],$$

with $p\left(x,0^{+}\right)=\delta \left(x\right)$. The equivalence of the two forms (88) and (89) is easily proved in the Fourier-Laplace domain by multiplying both sides of Equation (87) with the factor $s^{1-\beta }$.

We note that the choice (i) may be considered as a limit of the choice (ii) as β=1. In fact, in this limit we find $\tilde{H}\left(s\right)\equiv 1$ so $H\left(t\right)=t^{-1}/\Gamma \left(0\right)\equiv \delta \left(t\right)$ so that Equations (78)–(79) reduce to Equations (83)–(84), respectively. In this case the order of the Caputo derivative reduces to 1 and that of the R-L derivative to 0, whereas the Mittag-Leffler waiting time law reduces to the exponential.

In the sequel we will formally unite the choices (**i**) and (**ii**) by defining what we call the Mittag-Leffler memory function

(90) $$H^{ML}\left(t\right)=\left\{\begin{array}{cc} \frac{t^{-\beta }}{\Gamma (1-\beta )}\;, & \mathrm{if}\;0<\beta <1\;,\\ \delta (t)\;, & \mathrm{if}\;\beta =1\;, \end{array}\right.$$

whose Laplace transform is

(91) $${\tilde{H}}^{ML}\left(s\right)=s^{\beta -1}\;,\;0<\beta \leq 1\;.$$

Thus we will consider the whole range 0<β≤1 by extending the Mittag-Leffler waiting time law in (86) to include the exponential law (82).

**Remark** **1.** *Equation (79) clearly may be supplemented by an arbitrary initial probability density p(x,0)=f(x). The corresponding replacement of δ(x) by f(x) in (73) then requires in (76) multiplication of the term $(1-\tilde{\phi }\left(s\right))/s$ by $\hat{f}\left(\kappa \right)$ and in (78) replacement of the LHS by $\tilde{H}\left(s\right)\;\left[s\hat{\tilde{p}}\left(\kappa ,s\right)-\hat{f}\left(\kappa \right)\right]$. With p(x,0)=δ(x) we obtain in p(x,t) the fundamental solution of Equation (79).*

**Note:** The probability density function for the waiting time distribution in terms of the Mittag-Leffler function was formerly given since 1995 by Hilfer [55,56,57]. In these papers the waiting time density was given with the Mittag-Leffler function in two parameters without noting the relation with the first derivative of the classical Mittag-Leffler function as stated in Equation (29). We also note that 10 years earlier Balakrishnan [58] had derived a similar expression without recognizing the Mittag-Leffler function. Like in the case of the thinning process dealt by Gnedenko-Kowalenko (see Section 7) once again the Mitag-Leffler function was unknown for the authors.

### Manipulations: Rescaling and Respeeding

We now consider two types of manipulations on the CTRW by acting on its governing Equation (79) in its Laplace-Fourier representation (78).

(**A**): rescaling the waiting time, hence the whole time axis;

(**B**): respeeding the process.

(**A**) means change of the unit of time (measurement). We replace the random waiting time *T* by a waiting time τT, with the positive *rescaling factor* τ. Our idea is to take 0<τ≪1 in order to bring into near sight the distant future. In a moderate span of time we will so have a large number of jump events. For τ>0 we get the rescaled waiting time density

(92) $${\tilde{\phi }}_{\tau }\left(s\right)=\tilde{\phi }\left(\tau s\right)\;.$$

By decorating also the density *p* with an index τ we obtain the rescaled integral equation of the CTRW in the Laplace-Fourier domain as

(93) $${\tilde{H}}_{\tau }\left(s\right)\;\left[s{\hat{\tilde{p}}}_{\tau }\left(\kappa ,s\right)-1\right]=\left[\hat{w}\left(\kappa \right)-1\right]\;{\hat{\tilde{p}}}_{\tau }\left(\kappa ,s\right)\;,$$

where, in analogy to (77),

(94) $${\tilde{H}}_{\tau }\left(s\right)=\frac{1-\tilde{\phi }\left(\tau s\right)}{s\;\tilde{\phi }\left(\tau s\right)}\;.$$

(**B**) means multiplying the quantity representing $\frac{\partial }{\partial t}p\left(x,t\right)$ by a factor 1/a, where a>0 is the *respeeding factor*: a>1 means *acceleration*, 0<a<1 means *deceleration*. In the Laplace-Fourier representation this means multiplying the RHS of Equation (78) by the factor *a* since the expression $\left[s\hat{\tilde{p}}\left(\kappa ,s\right)-1\right]$ corresponds to $\frac{\partial }{\partial t}p\left(x,t\right)$.

We now chose to consider the procedures of rescaling and respeeding in their combination so that the equation in the transformed domain of the rescaled and respeeded process has the form

(95) $${\tilde{H}}_{\tau }\left(s\right)\;\left[s{\hat{\tilde{p}}}_{\tau ,a}\left(\kappa ,s\right)-1\right]=a\;\left[\hat{w}\left(\kappa \right)-1\right]\;{\hat{\tilde{p}}}_{\tau ,a}\left(\kappa ,s\right)\;,$$

Clearly, the two manipulations can be discussed separately: the choice {τ>0,a=1} means *pure rescaling*, the choice {τ=1,a>0} means *pure respeeding* of the original process. In the special case τ=1 we only respeed the original system; if 0<τ≪1 we can counteract the compression effected by rescaling to again obtain a moderate number of events in a moderate span of time by respeeding (decelerating) with 0<a≪1. These vague notions will become clear as soon as we consider power law waiting times.

Defining now

(96) $${\tilde{H}}_{\tau ,a}\left(s\right):=\frac{{\tilde{H}}_{\tau }\left(s\right)}{a}=\frac{1-\tilde{\phi }\left(\tau s\right)}{as\;\tilde{\phi }\left(\tau s\right)}\;.$$

we finally get, in analogy to (78), the equation

(97) $${\tilde{H}}_{\tau ,a}\left(s\right)\;\left[s{\hat{\tilde{p}}}_{\tau ,a}\left(\kappa ,s\right)-1\right]=\left[\hat{w}\left(\kappa \right)-1\right]\;{\hat{\tilde{p}}}_{\tau ,a}\left(\kappa ,s\right)\;.$$

What is the combined effect of rescaling and respeeding on the waiting time density?

In analogy to (77) and taking account of (96) we find

(98) $${\tilde{\phi }}_{\tau ,a}\left(s\right)=\frac{1}{1+s{\tilde{H}}_{\tau ,a}\left(s\right)}=\frac{1}{1+s\frac{1-\tilde{\phi }\left(\tau s\right)}{as\;\tilde{\phi }\left(\tau s\right)}}\;,$$

and so, for the deformation of the waiting time density, the *essential formula*

(99) $$\frac{a\;\tilde{\phi }\left(\tau s\right)}{1-\left(1-a\right)\tilde{\phi }\left(\tau s\right)}\;.$$

**Remark** **2.** *The formula (99) has the same structure as the thinning formula (66) in Section 5 (just devoted to the thinning theory) by identification of a with q. In both problems we have a rescaled process defined by a time scale τ, and we send the relevant factors τ, a and q to zero under a proper relationship. However in the thinning theory the relevant independent parameter going to 0 is that of thinning (actually respeeding) whereas in the present problem it is the rescaling parameter τ.*

## 9. Power Laws and Asymptotic Universality of the Mittag-Leffler Waiting Time Density

We have essentially two different situations for the waiting time distribution according to its first moment (the expectation value) being finite or infinite. In other words we assume for the waiting time pdfϕ(t) either

(100) $$\rho :=\int_{0}^{\infty }t^{\prime }\;\phi \left(t^{\prime }\right)\;dt^{\prime }<\infty \;,\;\mathrm{labelled}\;\mathrm{as}\;\beta =1\;,$$

or

(101) $$\phi \left(t\right)∼c\;t^{-(\beta +1)}\;\mathrm{for}\;t\to \infty \;\mathrm{hence}\;\;\Psi \left(t\right)∼\frac{c}{\beta }\;t^{-\beta }\;,\;0<\beta <1\;,\;c>0\;.$$

For convenience we have dispensed in (101) with decorating by a slowly varying function at infinity the asymptotic power law. Then, by the standard Tauberian theory (see References [21,48]) the above conditions (100)–(101) mean in the Laplace domain the (comprehensive) asymptotic form

(102) $$\tilde{\phi }\left(s\right)=1-\lambda s^{\beta }+o\left(s^{\beta }\right)\;\mathrm{for}\;s\to 0^{+}\;,\;0<\beta \leq 1\;,$$

where we have

(103) $$\lambda =\rho \;,\;\mathrm{if}\;\beta =1\;;\;\lambda =c\Gamma \left(-\beta \right)=\frac{c}{\Gamma (\beta +1)}\;\frac{\pi }{\sin(\beta \pi )}\;,\;\mathrm{if}\;0<\beta <1\;.$$

Then, *fixing s* as required by the continuity theorem of probability theory for Laplace transforms, taking

(104) $$a=\lambda \tau^{\beta }\;,$$

and *sending τ to zero*, we obtain in the limit the Mittag-Leffler waiting time law. In fact, Equations (99) and (102) imply as τ→0 with 0<β≤1,

(105) $${\tilde{\phi }}_{\tau ,\lambda \tau^{\beta }}\left(s\right)=\frac{\lambda \tau^{\beta }\;\left[1-\lambda \tau^{\beta }s^{\beta }+o\left(\tau^{\beta }s^{\beta }\right)\right]}{1-\left(1-\lambda \tau^{\beta }\right)\;\left[1-\lambda \tau^{\beta }s^{\beta }+o\left(\tau^{\beta }s^{\beta }\right)\right]}\to \frac{1}{1+s^{\beta }}\;,$$

the Laplace transform of $\phi^{ML}\left(t\right)$. This formula expresses **the asymptotic universality of the Mittag-Leffler waiting time law** that includes the exponential law for β=1. It can easily be generalized to the case of power laws decorated with slowly varying functions, thereby using the Tauberian theory by Karamata (see again References [21,48]).

**Comment:** The formula (105) says that our general power law waiting time density is gradually deformed into the Mittag-Leffler waiting time density as τ tends to zero.

**Remark** **3.** *Let us stress here the distinguished character of the Mittag-Leffler waiting time density $\phi^{ML}\left(t\right)=-\frac{d}{dt}E_{\beta }\left(-t^{\beta }\right)$. Considering its Laplace transform*

(106) $${\tilde{\phi }}^{ML}\left(s\right)=\frac{1}{1+s^{\beta }}\;,\;\phi^{ML}\left(t\right)=-\frac{d}{dt}E_{\beta }\left(-t^{\beta }\right)\;,\;0<\beta \leq 1\;,$$

*we can easily prove the identity*

(107) $${\tilde{\phi }}_{\tau ,a}^{ML}\left(s\right)={\tilde{\phi }}^{ML}\left(\tau s/a^{1/\beta }\right)\;for\;all\;\tau >0,\;a>0\;.$$

Note that Equation (107) states the *self-similarity* of the combined operation *rescaling-respeeding* for the Mittag-Leffler waiting time density. In fact, (107) implies $\phi_{\tau ,a}^{ML}\left(t\right)=\phi^{ML}\left(t/c\right)/c$ with $c=\tau /a^{1/\beta }\;,$ which means replacing the random waiting time $T^{ML}$ by $c\;T^{ML}$. As a consequences, choosing $a=\tau^{\beta }$ we have

(108) $${\tilde{\phi }}_{\tau ,\tau^{\beta }}^{ML}\left(s\right)={\tilde{\phi }}^{ML}\left(s\right)\;\mathrm{for}\;\mathrm{all}\;\tau >0\;.$$

Hence *the Mittag-Leffler waiting time density is invariant against combined rescaling with τ and respeeding with $a=\tau^{\beta }$*.

Observing (105) we can say that $\phi^{ML}\left(t\right)$ is a τ→0 attractor for any power law waiting time (101) under simultaneous rescaling with τ and respeeding with $a=\lambda \tau^{\beta }$. In other words, this attraction property of the Mittag-Leffler probability distribution with respect to power law waiting times (with 0<β≤1) is a kind of analogy to the attraction of sums of power law jump distributions by stable distributions.

## 10. The Mittag-Leffler Functions W.R.T the Time Fractional Diffusion-Wave Equations and the Wright Functions

In this section we show the relations of the Mittag-Leffler function with the Wright function via Laplace and Fourier transformations, in order to provide other arguments to outline the role of the Mittag-Leffler in the Fractional Calculus. For this purpose, because of the necessity to work with two independent parameters we first recall the proper definitions of the Mittag-Leffler and the Wright function. Then we will consider the time fractional diffusion-wave equation with its fundamental solutions to the basic boundary value problem that urn out to be expressed in terms of some special cases of the Wright functions, the so called *F* and *M* functions. Finally we pay attention to some noteworthy formulas for the *M*-Wright function, including its connections with the Mittag-Leffler function.

### 10.1. Definitions and Main Properties of the Wright Functions

The classical *Wright function*, that we denote by $W_{\lambda ,\mu }\left(z\right)$, is defined by the series representation convergent in the whole complex plane,

(109) $$W_{\lambda ,\mu }\left(z\right):=\sum_{n=0}^{\infty }\frac{z^{n}}{n!\Gamma (\lambda n+\mu )},\;\;\;\lambda >-1,\;\;\;\mu \in C,$$

As a consequence $W_{\lambda ,\mu }\left(z\right)$ is an *entire function* for all λ∈(−1,+∞). Originally Wright assumed λ≥0 in connection with his investigations on the asymptotic theory of partition [59,60] and only in 1940 he considered −1<λ<0, [61]. We note that in the Vol 3, Chapter 18 of the handbook of the Bateman Project [6], presumably for a misprint, the parameter λ is restricted to be non-negative, whereas the Wright functions remained practically ignored in other handbooks. In 1993 the present author, being aware only of the Bateman handbook, proved that the Wright function is entire also for −1<λ<0 in his approaches to the time fractional diffusion equation, as outlined in his papers published from 1994 to 1997, [62,63,64,65,66]. For other earlier treatments of this function we refer to the 1999 paper by Gorenflo, Luchko and Mainardi [67]).

In view of the asymptotic representation in the complex domain and of the Laplace transform the Wright functions were distinguished by the author in *first kind* (λ≥0) and *second kind* (−1<λ<0) as outlined e.g., in the Appendix F of his book [22].

We note that the Wright functions are entire of order 1/(1+λ) hence only the first kind functions (λ≥0) are of exponential order whereas the second kind functions (−1<λ<0) are not of exponential order. The case λ=0 is trivial since $W_{0,\mu }\left(z\right)=e^{z}/\Gamma (\mu ).$

Following the profs in the Appendix F in Reference [22] we get the following Laplace transform pairs of the Wright functions in terms of the Mittag-Leffler functions in two parameters, where *r* can be the time variable t>0 or the space variable x>0)

*for the first kind* (λ≥0)

(110) $$W_{\lambda ,\mu }\left(\pm r\right)\;÷\;\frac{1}{s}\;E_{\lambda ,\mu }\left(\pm \frac{1}{s}\right)\;,\;\lambda >0\;,$$

*for the second kind* (λ=−ν,0<ν<1)

(111) $$W_{-\nu ,\mu }\left(-r\right)\;÷\;E_{\nu ,\mu +\nu }\left(-s\right)\;,\;0<\nu <1\;.$$

The Wright functions of the first kind are useful to find the solutions of some (linear and non-linear) differential equations of fractional order as recently shown by Garra and Mainardi, [68].

Since the pioneering works in 1990’s by the author, noteworthy cases of Wright functions of the second kind, known as *auxiliary functions* *F* and *M* play fundamental roles in solving the Signalling problem and the Cauchy value problem, respectively for the time fractional diffusion-wave equation.

We first recall hereafter these auxiliary functions in terms of the Wright functions of the second kind, following their power series representations. They read

(112) $$F_{\nu }\left(z\right):=W_{-\nu ,0}\left(-z\right)\;,\;0<\nu <1\;,$$

and

(113) $$M_{\nu }\left(z\right):=W_{-\nu ,1-\nu }\left(-z\right)\;,\;0<\nu <1\;,$$

interrelated through

(114) $$F_{\nu }\left(z\right)=\nu \;z\;M_{\nu }\left(z\right)\;.$$

The *series representations* of our auxiliary functions are derived from those of $W_{\lambda ,\mu }\left(z\right)$ in (109). We have:

(115) $$F_{\nu }\left(z\right)=\sum_{n=1}^{\infty }\frac{{\left(-z\right)}^{n}}{n!\;\Gamma (-\nu n)}=-\frac{1}{\pi }\;\sum_{n=1}^{\infty }\frac{{\left(-z\right)}^{n}}{n!}\;\Gamma \left(\nu n+1\right)\;\sin\left(\pi \nu n\right)\;,$$

and

(116) $$M_{\nu }\left(z\right)=\sum_{n=0}^{\infty }\frac{{\left(-z\right)}^{n}}{n!\;\Gamma [-\nu n+(1-\nu )]}=\frac{1}{\pi }\;\sum_{n=1}^{\infty }\;\frac{{\left(-z\right)}^{n-1}}{(n-1)!}\;\Gamma \left(\nu n\right)\;\sin\left(\pi \nu n\right)\;,$$

where we have used the well-known reflection formula for the Gamma function,

Γ(ζ)Γ(1−ζ)=π/sinπζ.

### 10.2. The Time-Fractional Diffusion-Wave Equation and the Related Green Functions

For the reader’s convenience let us recall the main formulas for the time fractional diffusion equations and their fundamental solutions (also referred to as the Green functions) for the Cauchy and Signalling problems. For more details we refer to References [69,70].

Denoting as usual x,t the space and time variables, and r=r(x,t) the response variable, the family of these evolution equations reads

(117) $$\frac{\partial^{\beta }r}{\partial t^{\beta }}=a\;\frac{\partial^{2}r}{\partial x^{2}}\;,\;0<\beta \leq 2\;,$$

where *the time derivative of order β is intended in the Caputo sense*, namely is the operator ${\;}_{*}D_{t}^{\beta }$, introduced in Section 3, but for order less than 1, see Equation (10), and *a* is a positive constant of dimension $L^{2}\;T^{-\beta }$. Thus we must distinguish the cases 0<β≤1 and 1<β≤2. We have

(118) $$\frac{\partial^{\beta }r}{\partial t^{\beta }}:=\left\{\begin{array}{cc} \frac{1}{\Gamma (1-\beta )}\;\int_{0}^{t}\;\;\left[\frac{\partial }{\partial \tau }\;r\left(x,\tau \right)\right]\;\frac{d\tau }{{\left(t-\tau \right)}^{\beta }}\;, & \;0<\beta <1\;,\\ \frac{\partial r}{\partial t}\;, & \;\beta =1\;; \end{array}\right.$$

(119) $$\frac{\partial^{\beta }r}{\partial t^{\beta }}\;:=\;\left\{\begin{array}{cc} \frac{1}{\Gamma (2-\beta )}\int_{0}^{t}\;\;\left[\frac{\partial^{2}}{\partial \tau^{2}}\;r\left(x,\tau \right)\right]\;\frac{d\tau }{{\left(t-\tau \right)}^{\beta -1}}, & 1<\beta <2,\\ \frac{\partial^{2}r}{\partial t^{2}}\;, & \beta =2\;. \end{array}\right.$$

It should be noted that in both cases 0<β≤1,1<β≤2, the time fractional derivative in the L.H.S. of Equation (117) can be removed by a suitable fractional integration. leading to alternative forms where the necessary initial conditions at $t=0^{+}$ explicitly appear.

For this purpose we apply to Equation (117) the fractional integral operator of order β, namely

$$J_{t}^{\beta }f\left(t\right):=\frac{1}{\Gamma (\beta )}\;\int_{0}^{t}{\left(t-\tau \right)}^{\beta -1}\;f\left(\tau \right)\;d\tau .$$

For β∈(0,1] we have:

$$J_{t}^{\beta }\;\circ {\;}_{*}D_{t}^{\beta }\;r\left(x,t\right)=\;J_{t}^{\beta }\;\circ \;J_{t}^{1-\beta }\;D_{t}^{1}\;r\left(x,t\right)=\;J_{t}^{1}\;D_{t}^{1}\;r\left(x,t\right)=r\left(x,t\right)-r\left(x,0^{+}\right)\;.$$

For β∈(1,2] we have:

$$J_{t}^{\beta }\;\circ {\;}_{*}D_{t}^{\beta }\;r\left(x,t\right)\;=\;\;J_{t}^{\beta }\;\circ \;I_{t}^{2-\beta }\;D_{t}^{2}\;r\left(x,t\right)\;=\;\;J_{t}^{2}\;D_{t}^{2}\;r\left(x,t\right)\;=\;r\left(x,t\right)-r\left(x,0^{+}\right)-t\;r_{t}\left(x,0^{+}\right).$$

Then, as a matter fact, we get the integro-differential equations:

if 0<β≤1:

(120) $$r\left(x,t\right)=r\left(x,0^{+}\right)+\frac{a}{\Gamma (\beta )}\;\int_{0}^{t}\;\;\left(\frac{\partial^{2}r}{\partial x^{2}}\right)\;{\left(t-\tau \right)}^{\beta -1}\;d\tau \;;$$

if 1<β≤2:

(121) $$r\left(x,0^{+}\right)+t\;\frac{\partial }{\partial t}{\left.r(x,t)\right|}_{t=0^{+}}+\frac{a}{\Gamma (\beta )}\int_{0}^{t}\;\;\left(\frac{\partial^{2}r}{\partial x^{2}}\right){\left(t-\tau \right)}^{\beta -1}\;d\tau .$$

Denoting by f(x),x∈IR and $h\left(t\right)\;,\;t\in {I\;R}^{+}\;$ sufficiently well-behaved functions, the basic boundary-value problems are thus formulated as following, assuming 0<β≤1,

*(a) Cauchy problem*

(122) $$r\left(x,0^{+}\right)=f\left(x\right)\;,\;-\infty <x<+\infty \;;\;r\left(\mp \infty ,t\right)=0\;,\;\;t>0\;;$$

*(b) Signalling problem*

(123) $$r\left(x,0^{+}\right)=0\;,\;x>0\;;\;r\left(0^{+},t\right)=h\left(t\right)\;,\;r\left(+\infty ,t\right)=0\;,\;t>0\;.$$

If 1<β<2, we must add into (122) and (123) the initial values of the first time derivative of the field variable, $r_{t}\left(x,0^{+}\right)\;,$ since in this case the corresponding fractional derivative is expressed in terms of the second order time derivative. To ensure the continuous dependence of our solution with respect to the parameter β also in the transition from $\beta =1^{-}$ to $\beta =1^{+}\;,$ we agree to assume

(124) $$\frac{\partial }{\partial t}{\left.r(x,t)\right|}_{t=0^{+}}=0\;,\;\mathrm{for}\;1<\beta \leq 2\;,$$

as it turns out from the integral forms (120)–(121).

In view of our subsequent analysis we find it convenient to set

(125) $$\nu :=\beta /2\;,\;\mathrm{so}\;\left\{\begin{array}{c} 0<\nu \leq 1/2\;,\;⟺\;0<\beta \leq 1\;,\\ 1/2<\nu \leq 1\;,\;⟺\;1<\beta \leq 2\;, \end{array}\right.$$

and from now on to add the parameter ν to the independent space-time variables x,t in the solutions, writing r=r(x,t;ν).

For the Cauchy and Signalling problems we introduce the so-called *Green functions* $G_{c}\left(x,t;\nu \right)$ and $G_{s}\left(x,t;\nu \right)$, which represent the respective fundamental solutions, obtained when f(x)=δ(x) and h(t)=δ(t). As a consequence, the solutions of the two basic problems are obtained by a space or time convolution according to

(126) $$r\left(x,t;\nu \right)=\int_{-\infty }^{+\infty }G_{c}\left(x-\xi ,t;\nu \right)\;f\left(\xi \right)\;d\xi \;,$$

(127) $$r\left(x,t;\nu \right)=\int_{0^{-}}^{t^{+}}G_{s}\left(x,t-\tau ;\nu \right)\;h\left(\tau \right)\;d\tau \;.$$

It should be noted that in (126) $G_{c}\left(x,t;\nu \right)=G_{c}\left(|x|,t;\nu \right)$ because the Green function of the Cauchy problem turns out to be an even function of *x*. According to a usual convention, in (127) the limits of integration are extended to take into account for the possibility of impulse functions centred at the extremes.

Now we recall the results obtained in 1990’s by the author that allow us to express the two Green functions in terms of the auxiliary functions $F_{\nu }\left(\xi \right)$ and $M_{\nu }\left(\xi \right)$ where, for x>0, t>0

(128) $$\xi :=x/(\sqrt{a}\;t^{\nu })>0$$

acts as *similarity variable*. Then we obtain the Green functions in the space-time domain in the form

(129) $$G_{c}\left(x,t;\nu \right)=\frac{1}{2\;\nu \;x}\;F_{\nu }\left(\xi \right)=\frac{1}{2\sqrt{a}\;t^{\nu }}\;M_{\nu }\left(\xi \right)\;,$$

(130) $$G_{s}\left(x,t;\nu \right)=\frac{1}{t}\;F_{\nu }\left(\xi \right)=\frac{\nu \;x}{\sqrt{a}\;t^{1+\nu }}\;M_{\nu }\left(\xi \right)\;.$$

We also recognize the following *reciprocity relation* for the original Green functions,

(131) $$2\nu \;x\;G_{c}\left(x,t;\nu \right)=t\;G_{s}\left(x,t;\nu \right)=F_{\nu }\left(\xi \right)=\nu \xi \;M_{\nu }\left(\xi \right)\;.$$

Now $F_{\nu }\left(\xi \right)$, $M_{\nu }\left(\xi \right)$ are the *auxiliary functions* for the general case 0<ν≤1, which generalize those well known for the standard (Fourier) diffusion equation and for the standard (D’alembert) wave equation derived for ν=1/2 and for ν=1, respectively.

### 10.3. Some Noteworthy Results for the $M_{\nu }$ Wright Function

In this survey we find worthwhile to concentrate our attention on a single auxiliary function, the *M*-Wright function, sometimes referred to as the *Mainardi function*. Indeed this function is indeed referred with this name in n the 1999 book by Podlubny [19], that is one of the most cited treatises on fractional calculus. Then this name is found in several successive papers and books related to fractional diffusion and wave processes, see for example, the relevant 2015 paper by Sandev et al. [71].

Let us now recall some interesting analytic results related to the so-called Mainardi function. One reason for the major attention is due to its straightforward generalization of the Gaussian probability density obtained for ν=1/2, that is the fundamental solution of the Cauchy problem for the standard diffusion equation. Furthermore it allows an impressive visualization of the evolution with the order ν∈(0,1) of the Green function of the Cauchy problem of the fractional diffusion wave Equation (129) as shown in the next figures with a=1 and taking t=1.

**Figure 6:**
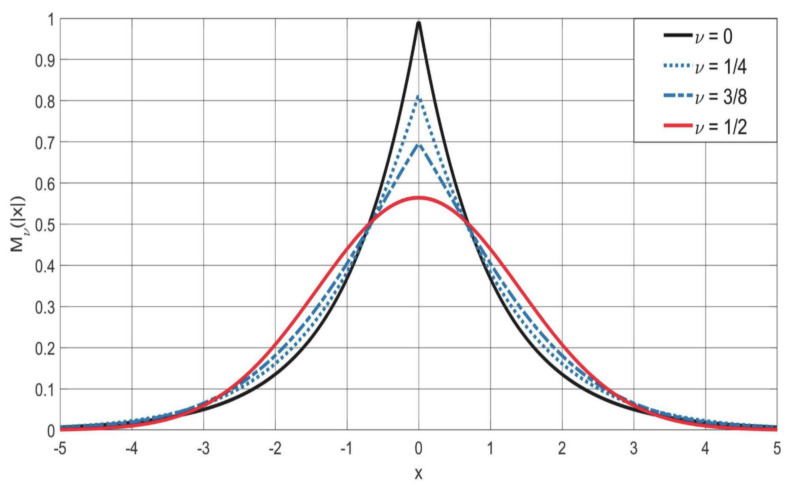
Plot of the symmetric M−Wright function $M_{\nu }\left(|x|\right)$ for 0≤ν≤1/2. Note that the M−Wright function becomes a Gaussian density for ν=1/2.

**Figure 7:**
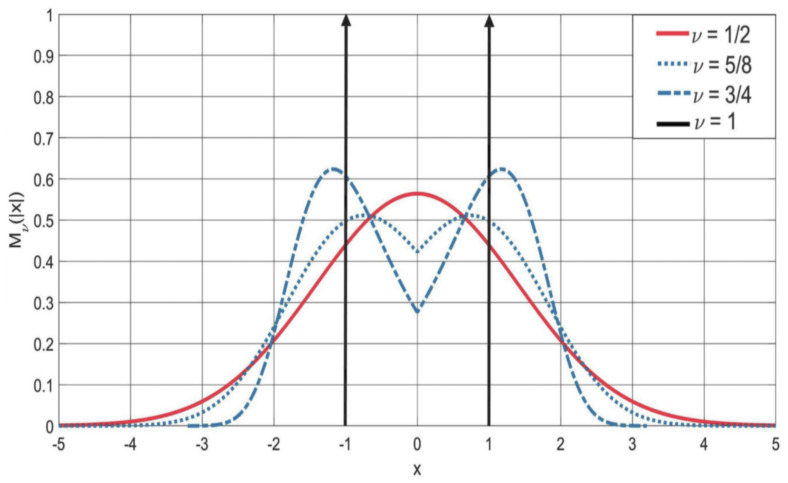
Plot of the symmetric M−Wright type function $M_{\nu }\left(|x|\right)$ for 1/2≤ν≤1. Note that the *M*Wright function becomes a a sum of two delta functions centered in x=±1 for ν=1.

The readers are invited to look the YouTube video by my former student Armando Consiglio whose title is “Simulation of the M−Wright function”, in which the author has shown the evolution of this function as the parameter ν changes between 0 and 0.85 in the interval (−5<x<+5) of IR centered in x=0 represented herewith in Figure 6 and Figure 7 at fixed time t=1.

The readers interested to have more details on the classical Wright functions would consult the recent survey by Luchko [72] and references therein.

In view of time-fractional diffusion processes related to time-fractional diffusion equations it is worthwhile to introduce the function in two variables

(132) $$M_{\nu }\left(x,t\right):=t^{-\nu }\;M_{\nu }\left(xt^{-\nu }\right)\;,\;0<\nu <1\;,\;x,t\in {I\;R}^{+}\;,$$

which defines a spatial probability density in *x* evolving in time *t* with self-similarity exponent H=ν. Of course for x∈IR we have to consider the symmetric version of the *M*-Wright function. obtained from (132) multiplying by 1/2 and replacing *x* by |x|.

Hereafter we provide a list of the main properties of this function, which can be derived from the Laplace and Fourier transforms for the corresponding Wright *M*-function in one variable presented in papers by Mainardi and recalled in the Appendix F of Reference [22].

For the Laplace transform of $M_{\nu }\left(x,t\right)$ with respect to t>0 and x>0 we get respectively:

(133) $$L\left\{M_{\nu }\left(x,t\right);t\to s\right\}:=\int_{0}^{\infty }e^{-st}\;t^{-\nu }\;M_{\nu }\left(x\;t^{-\nu }\right)\;dt=s^{\nu -1}\;e^{\;-xs^{\nu }}\;;$$

(134) $$L\left\{M_{\nu }\left(x,t\right);x\to s\right\}:=\int_{0}^{\infty }e^{-sx}\;t^{-\nu }\;M_{\nu }\left(x\;t^{-\nu }\right)\;dx=E_{\nu ,1}\left(-st^{\nu }\right)\;.$$

For the Fourier transforms with respect to the spatial variable *x* we have for $M_{\nu }\left(x,t\right)$ with $x\in {I\;R}^{+}$,

(135) $$\begin{array}{cc} & F_{C}\left\{M_{\nu }\left(x,t\right);x\to \kappa \right\}:=\int_{0}^{\infty }\cos\left(\kappa x\right)\;t^{-\nu }\;M_{\nu }\left(x\;t^{-\nu }\right)\;dx=E_{2\nu ,1}\left(-\kappa^{2}t^{2\nu }\right)\;,\\ & F_{S}\left\{M_{\nu }\left(x,t\right);x\to \kappa \right\}:=\int_{0}^{\infty }\sin\left(\kappa x\right)\;t^{-\nu }\;M_{\nu }\left(x\;t^{-\nu }\right)\;dx=\kappa^{\nu }\;E_{2\nu ,\nu +1}\left(-\kappa^{2}t^{2\nu }\right)\;, \end{array}$$

so that for the symmetric function $M_{\nu }\left(|x|,t\right)$ we get

(136) $$F\left\{M_{\nu }\left(|x|,t\right);x\to \kappa \right\}=2\int_{0}^{\infty }\cos\left(\kappa x\right)\;t^{-\nu }\;M_{\nu }\left(x\;t^{-\nu }\right)\;dx=2E_{2\nu ,1}\left(-\kappa^{2}t^{2\nu }\right)\;.$$

Restricting our attention at the known analytic expressions of the $M_{\nu }$ functions versus *x* at fixed time t=1 we recall the following results for some special rational values of the parameter ν:

ν=1/3 (see Reference [22])

(137) $$M_{1/3}\left(x\right)=3^{2/3}\mathrm{Ai}\left(x/3^{1/3}\right)\;,$$

ν=1/2 (see Reference [22])

(138) $$M_{1/2}\left(x\right)=\frac{1}{\sqrt{\pi }}e^{-x^{2}/4}\;,$$

ν=2/3 (see Reference [73])

(139) $$M_{2/3}\left(x\right)=3^{-2/3}\left[3^{1/3}\;x\;\mathrm{Ai}\left(x^{2}/3^{4/3}\right)-3{\mathrm{Ai}}^{\prime }\left(x^{2}/3^{4/3}\right)\right]\;e^{-2x^{3}/27}\;.$$

In the above equations Ai and ${\mathrm{Ai}}^{\prime }$ denote the *Airy function* and its first derivative.

## Figures and Tables

**Figure A1:**
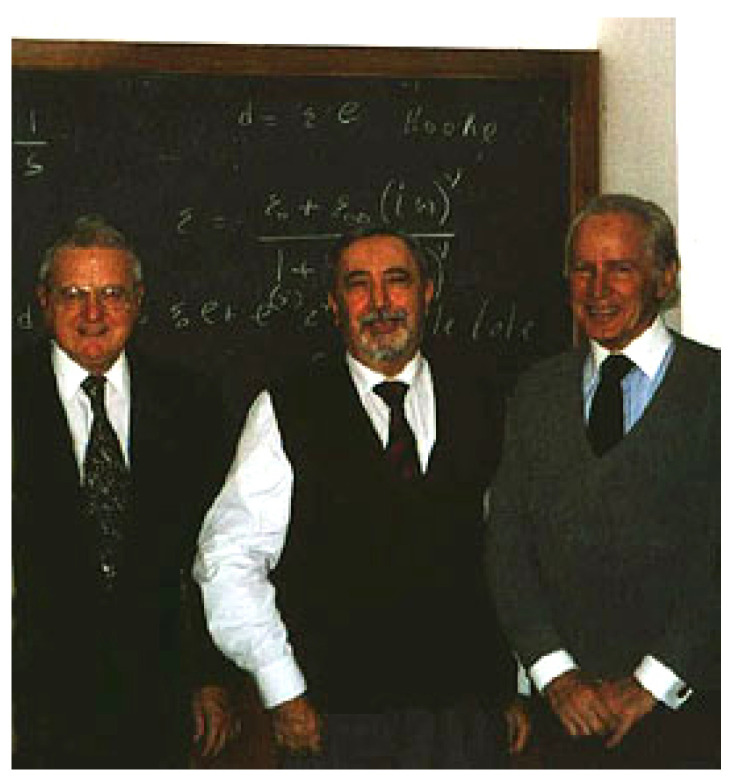
F. Mainardi between R. Gorenflo (left) and M. Caputo (right).

## Appendix A. My Acquaintance with the Mittag-Leffler Function Since the Late 1960’s

I was formerly acquainted with the Mittag-Leffler function from the pioneering 1947 paper by Gross on creep and relaxation in linear viscoelasticity. It was during my PhD sties at the University of Bologna under the supervision of Prof Caputo in the year 1969. Indeed I was asked to apply in the framework of anelastic materials the derivative of non-integer order introduced by Prof Caputo in [74,75]. More recently this fractional derivative was named after him thanks the suggestions of Gotrenflo and Mainardi [18] and Podlubny [19]. I understood that the Mittag-Leffler function proposed by Gross both in creep and relaxation processes could be used in the corresponding processes in the fractional Zener model. Because Gross had computed and plotted only the spectra, see Figure 1 in this article, I was interested to plot the Mittag-Leffler function on which I was addressed in the Third volume of the Handbook of the Bateman Project published in 1955 [6]. Carrying out the plot of the Mittag-Leffler function $E_{\alpha }\left(-t^{\alpha }\right)$ using a Fortran program was not easy for me using its power series representation, so I limited the time interval to [0.5] with ordinate in logarithmic scale. As far as I know this was the former plot of this function, see References [9,10] where the results of my PhD thesis were published in 1971 jointly with my supervisor. Later I was acquainted with the viscoelastic model by Rabotnov in 1948 [76] and with the Russian school of Meskov and Rossikhin who used the so-called Rabotnov function, indeed related to the Mittag-Leffler function. and consequently with results similar to some extent to those in References [9,10]. However, our work was totally independent from the Russian school (incidentally published in Russian), as outlined in the Notes to the chapter 3 of my 2010 book, see pp. 74–76 in [22]. More later, in the 1980 I was acquainted with the results by Bagley-Torvik and by Koeller that confirmed the relevant role of the Mittag-Leffler functions in linear viscoelastic models governed by constitutive laws of fractional order. Once again their results crossed with those in References [9,10]. However, I have to confess that, when in conferences of those years I dealt with fractional derivatives in rheology, the audience remained indifferent if not hostile and laughable so I left this topic preferring to transfer my research interests to wave phenomena, in particular on the effects of dissipation on linear dispersive waves.

Incidentally, in 1980’s, I was also aware of the nice treatise by Harold T. Davis on the Theory of Linear operators published in 1936 [2], where the author gave information about the fractional calculus and the Mittag-Leffler function. It was my honor to publish a recent survey on the contributions by Davis and Gross (already recalled in Introduction), whom I consider the pioneers of fractional relaxation processes in viscoelastic and dielectric materials [32]. In the firsts years of 1990s under the push of fractals, the relevance of fractional derivatives (used not always in a correct way) was outlined in several papers. For this I was induced to come back to fractional calculus. It was just this occasion for me to devote my research interests to the application of fractional calculus in relaxation, oscillation phenomena governed by fractional ODEs and diffusion, wave phenomena governed by fractional PDEs. Once again I understood the relevance of the Mittag-Leffler functions but also that of the Wright functions, both of them classified as miscellaneous functions in the handbook of Bateeman project. I must note that, as far as I known, the Bateman handbook was the only one published in English to deal with these special function, and therefore accessible to me.

The year 1994 was the golden year for me as far as my acquaintance with fractional calculus and related special functions is concerned. Indeed I took profit by the acquaintance in three different conferences with the late Prof Gorenflo and Prof. Nigmatullin (in Bordeaux, France), with Prof. Podlubny and Prof. Caputo (in Astlanta, USA), and with Prof Virginia Kiryakova and the late Prof. Stankovic (in Sofia, Bulgaria), among other authorities of the fractional calculus. But it was with Prof Gorenflo that I started a collaboration for more than 20 years (1995–2015) motivated by our common interest towards the potential of the Mittag-Leffler functions in the applications of the fractional calculus.

Then, since 1997, I was interested in the emerging science of Econophysics thanks mainly to my younger colleague Enrico Scalas. With Gorenflo, Scalas and his student Raberto we published some papers on the advent of fractional Calculus in Econophysics, see e.g., [54] and my historical survey in Mathematics [77]. In 2007, on the occasion of the 80-birthday of Prof. Caputo, I published with Gorenflo a survey in Fractional Calculus and Applied Analysis [11] where I took the liberty to propose for the Mittag-Leffler function the (successful) title of *the Queen Function of the Fractional Calculus*. Some years earlier, Gorenflo had contacted the American Mathematical Society to give a specific number to the Mittag-Leffler function, that is 33E12, in the MSC classification.

Gorenflo and I promoted the Mittag-Lffler functions in several Conferences and Workshops in all the world. In particular, I would like to recall my lectures in India (under invitation of Prof Mathai, director of the Center of Mathematical Sciences, in Brazil (under invitation of Prof Edmundo Capelas de Oliveira, Campinas University) and in US (under invitation of Prof. Karniadakis, Brown University, see Reference [78]).

I like to outline my gratitude to Professor Michele Caputo (1927) and Rudolf Gorenflo (1930–2017) for having provided me with useful advice in earlier and later times, respectively. It is my pleasure to enclose a photo showing the author between them, taken in Bologna, April 2002.

Unfortunately, I lost Gorenflo’s guidance and collaboration in 2015 when he suffered strong health troubles that led him to his death on 20 October 2017 at 87 years. He was Emeritus Professor of Mathematics at the Free University of Berlin since his retirement in 1998.

Nowadays I am quite interested to promote the special functions of the Mittag-Leffler and Wright type with the second edition of the treatise by Gorenflo et al. [14] and my surveys [79,80], including the present review.
